# A Generic System for the Expression and Purification of Soluble and Stable Influenza Neuraminidase

**DOI:** 10.1371/journal.pone.0016284

**Published:** 2011-02-07

**Authors:** Peter M. Schmidt, Rebecca M. Attwood, Peter G. Mohr, Susan A. Barrett, Jennifer L. McKimm-Breschkin

**Affiliations:** 1 Division of Materials Science & Engineering, CSIRO, Parkville, Victoria, Australia; 2 Australian Animal Health Laboratory, CSIRO, Geelong, Victoria, Australia; University Paris Diderot-Paris 7, France

## Abstract

The influenza surface glycoprotein neuraminidase (NA) is essential for the efficient spread of the virus. Antiviral drugs such as Tamiflu (oseltamivir) and Relenza (zanamivir) that inhibit NA enzyme activity have been shown to be effective in the treatment of influenza infections. The recent ‘swine flu’ pandemic and world-wide emergence of Tamiflu-resistant seasonal human influenza A(H1N1) H_274_Y have highlighted the need for the ongoing development of new anti-virals, efficient production of vaccine proteins and novel diagnostic tools. Each of these goals could benefit from the production of large quantities of highly pure and stable NA. This publication describes a generic expression system for NAs in a baculovirus Expression Vector System (BEVS) that is capable of expressing milligram amounts of recombinant NA. To construct NAs with increased stability, the natural influenza NA stalk was replaced by two different artificial tetramerization domains that drive the formation of catalytically active NA homotetramers: GCN4-pLI from yeast or the Tetrabrachion tetramerization domain from *Staphylothermus marinus*. Both recombinant NAs are secreted as FLAG-tagged proteins to allow for rapid and simple purification. The Tetrabrachion-based NA showed good solubility, increased stability and biochemical properties closer to the original viral NA than the GCN4-pLI based construct. The expressed quantities and high quality of the purified recombinant NA suggest that this expression system is capable of producing recombinant NA for a broad range of applications including high-throughput drug screening, protein crystallisation, or vaccine development.

## Introduction

Human seasonal influenza is responsible for a world-wide death toll of an estimated 250,000–500,000 and 3–5 million cases of severe illness routinely every year (WHO influenza fact sheet 2009), a fact easily forgotten in light of the media presence during the rapid spread of the ‘swine flu’ virus (human A(H1N1)-2009), the first influenza pandemic in more than 4 decades. In the wake of this newly emerged reassortant virus, the potential problems associated with rapidly producing sufficient doses of vaccine for millions of people became evident and highlighted the ongoing need to improve the production of immunogenic proteins.

One of the major influenza proteins that represents an important component of killed split vaccines is the viral neuraminidase (NA). In its active form NA is a membrane bound homotetrameric glycoprotein and its sialidase activity is essential for the release of new virus particles from infected cells[1]. Inhibition of NA activity by ethyl (3R,4R,5S)-5-amino-4-acetamido-3-(pentan-3-yloxy)cyclohex-1-ene-1-carboxylate (Tamiflu, oseltamivir) and (2R,3R,4S)- 4-[(diaminomethylidene)amino]- 3-acetamido- 2-[(1R,2R)- 1,2,3-trihydroxypropyl]- 3,4-dihydro- 2H-pyran- 6-carboxylic acid (Relenza, zanamivir) has proven to be effective in the treatment of influenza infections[1], [2]. However, NA inhibitor-resistant virus strains have recently emerged such as the A(H1N1) seasonal human influenza strain with an H_274_Y mutation, which confers resistance to Tamiflu [3], [4], [5]. Therefore, understanding the mechanisms behind antiviral resistance and the continual development of new antivirals based on that insight is critically important. Protein crystallisation has been a powerful approach to explain mechanisms of resistance, however the production of large amounts of highly purified NA has been problematic. Methods based on the amplification of influenza virus in eggs[6], [7] or cell culture systems rely on the viability of the virus. Often mutations that confer resistance have been shown to compromise virus viability resulting in extremely low levels of purified NA being obtained[8], [9]. In addition, proteases have been commonly used to cleave the NA head from the membrane[6],[7] or detergents were applied to solubilise the enzyme out of the viral membrane[10]. However, viral NAs can become intrinsically unstable once they are cleaved from the membrane[11] and lose activity within hours and therefore impede any subsequent analysis.

Recent alternatives have included the production of recombinant NA in eukaryotic expression systems, for example yeast[12], [13] or insect cells[14], [15], [16]. Both systems have been limited to date by the low yield of tetrameric, active NA[12], [15] or a dependence on proteases to cleave the NA head from the membrane[16], [17]. Although the use of cleavage site-specific proteases (e.g. Thrombin) has overcome the problem of non-specific cleavage they still could render the stalk-less NA head prone to dissociation into non-active NA monomers or dimers. The generic expression system outlined in the present publication aims to overcome these obstacles by a combination of the melittin signalling peptide (MSP) secretion signal[18], FLAG tag[19] and the use of two artificial tetramerization domains from yeast (GCN4-pLI, [20], [21]) and the deep sea archae bacterium *Staphylothermus marinus* (Tetrabrachion, [22]). The latter has been shown to be stable at temperatures up to 130°C making it a potentially good choice to stabilize a tetrameric active form of secreted soluble NA[23]. We demonstrated that a recombinant human N1 NA with an artificial Tetrabrachion stalk is stable and shares more similarities with the original viral NA when compared to the same protein with the yeast stalk GCN4-pLI.

## Results

### Construction of a generic expression system

**Figure 1:**
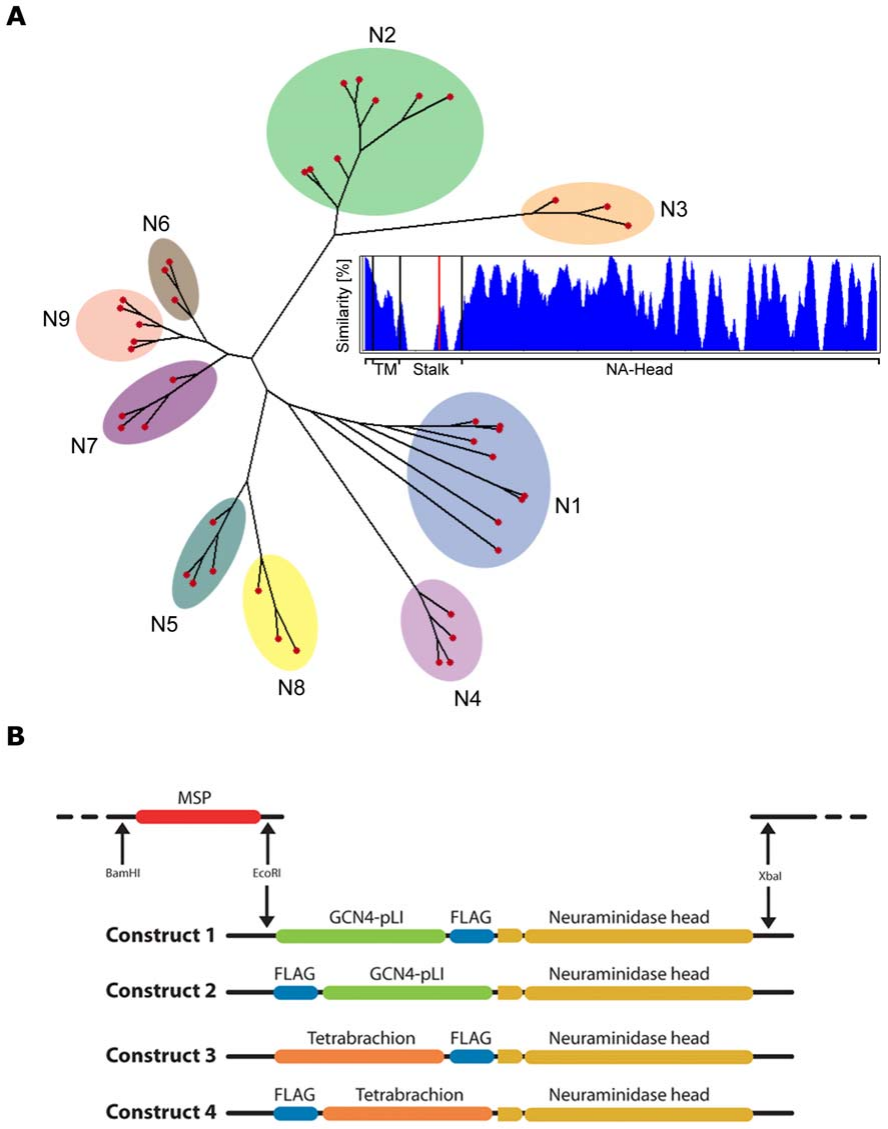
Construction of the expression system. (A) To identify potentially important domains in the NA stalk, 43 NA sequences covering N1 to N9 from 1918 to 2007 were included in the alignment (for details see Table S1). The inset shows the level of homology of the sequences for the NA domains, namely the cytoplasmatic domain, the transmembrane domain (TM), the stalk- and the head-domain. The alignment shows that although the majority of the stalk is not conserved a small stretch shows a higher degree of similarity. Amino acids 70–73 (based on N2 numbering) of this stretch were included in the expression system (the beginning of the region indicated by red line). (B) The sequences encoding for the 4 artificial NA constructs where cloned into pFastBac upstream from an MSP sequence using EcoRI and XbaI.

In order to allow the expression of a broad range of NAs and to identify conserved regions in the NA stalk domain, a multisequence alignment of 43 NA sequences ranging from N1 to N9 (1918–2007) was performed (for a complete list of included influenza strains see table S1). The result shows that although the NA stalk is highly variable, a stretch of 4 residues (70–73 based on N2 numbering) shows a higher degree of similarity (Fig. 1A). As it has previously been shown that the NA stalk can have an impact on NA catalytic activity [24], [25], [26], [27], these amino acids were included in the construction of the expression system, especially as they represent a predicted glycosylation site for various NAs. Sequences encoding the tetramerizing domains from yeast (transcription factor GCN4-pLI[21]) and *Staphylothermus marinus* (Tetrabrachion[22], [23]) were combined with an up- or downstream FLAG-tag resulting in 4 different artificial stalk constructs (Fig. 1B). The 4 artificial stalks were amplified, fused to the NA head of Hokkaido N1 by PCR and cloned into the Vector pFastBac downstream of a MSP secretion signal[28].

### Characterizing and optimizing the expression system

**Figure 2:**
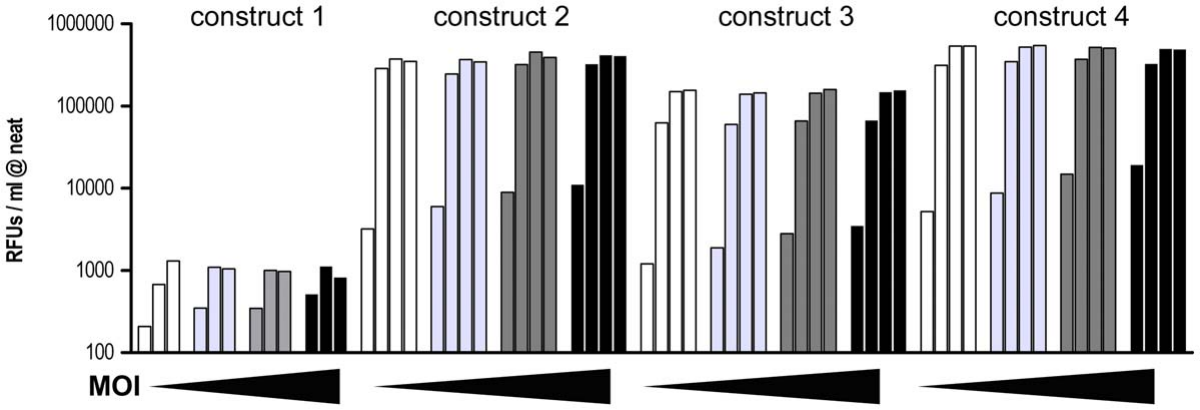
Optimizing MOI. To optimize the MOI, Sf21 cells were infected with all 4 constructs at a MOI of 1 (white bars), 2 (light gray bars), 3 (dark gray bars) and 4 (black bars). NA activity in the media was measured at 0 h, 24 h, 48 h and 72 h. Increasing the MOI did not have any significant impact on the amount of secreted NA activity. This experiment was performed once in duplicate.

Sf21 cells were infected with all 4 constructs at a Multiplicity of infection (MOI) of 1, 2, 3, and 4 and the respective NA activity in the media was measured after 0 h, 24 h, 48 h, and 72 h. As shown in figure 2, increasing the MOI above 1 did not result in increased expression levels of NA. All constructs but construct 1 showed strong NA activity in the media (Fig. 2) as well as strong signals in the corresponding anti-FLAG western blots (Figure S1). As construct 1 showed only negligible NA activity and low expression levels it was not further pursued.

**Figure 3:**
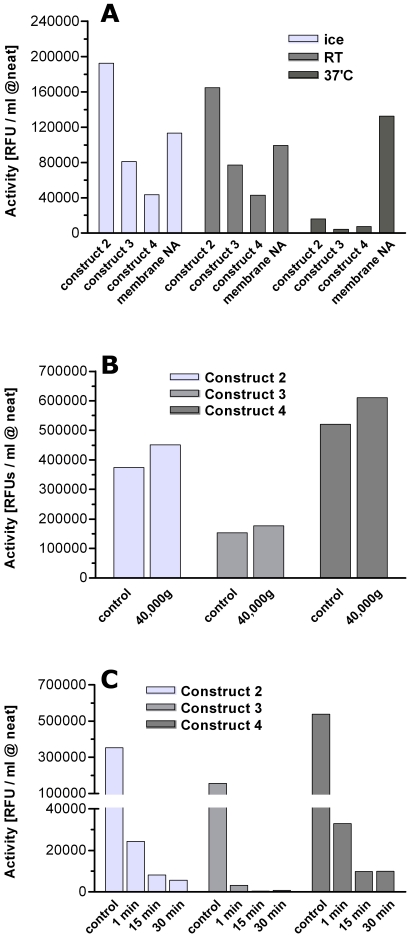
Characterization of secreted NA in media. (A) The different NA-containing media were incubated for 3 days on ice, RT, or 37°C. The secreted soluble construct 2–4 were stable at RT but not at 37°C whereas the membrane bound full length Hokkaido N1 was stable at all temperatures. (B) Centrifugation of construct 2–4 containing media for 1 h at 40,000 g did not affect the amount of detected NA activity. (C) Incubation of construct 2–4 containing media at pH 2.8 abolished most of the NA activity of construct 2–4 within 1 min. This experiment was performed once in duplicate.

Thermal stability studies showed that constructs 2–4 were stable at RT in SF900 II media for 3 days compared to control samples stored on ice (Fig. 3A). Incubation of the samples for this time at 37°C resulted in a strong decrease in the observed NA activity. The membrane-bound full length recombinant Hokkaido NA that was used as control was stable under these conditions. To demonstrate that the secreted NAs were truly soluble we showed that the NA activity in the supernatant remained unchanged after 1 h of centrifugation at 40,000 g (Fig. 3B).

Low pH is a convenient way to elute bound proteins from affinity columns and although the Tetrabrachion stalk has been reported to be stable at extreme pH[22], NA is known to lose its activity under these conditions[29], [30]. To determine if the artificial NAs were stable under low pH conditions, constructs 2–4 in SF 900II media were diluted in glycine buffer pH 2.8 for different times before measuring their activity. The data in figure 3C shows clearly that even a short-term incubation of 1 minute at low pH conditions reduced the NA activity by more than 90% (Fig. 3C) with construct 3 being more vulnerable than constructs 2 and 4.

**Figure 4:**
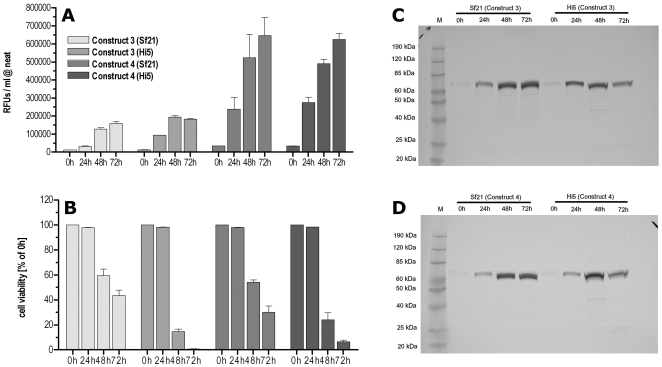
Comparing Sf21 and Hi5 insect cells. To optimize the expression system Sf21 cells were compared to Hi5 cells. (A) There was no significant difference in the amount of secreted NA activity observed for construct 3 or 4 in both cell lines. (B) Cell viability seemed to drop faster for Hi5 compared to Sf21 cells without affecting the amount of secreted NA activity. (C, D) Anti-FLAG western blot of construct 3- or 4-containing media showed a strong signal for both cell lines. Activity and viability data are duplicates of three independent infections. For the respective western blots the supernatants of all three infections were pooled.

In order to further optimize the expression system, Sf21 (*Spodoptera frugiperda)* cells were compared to Hi5 (*Trichopulsia ni*) insect cells as the latter cell line has been reported to give higher yields especially of secreted proteins. Both cell lines were infected with baculoviruses encoding for constructs 3 or 4 at a MOI of 1 and the secreted NA activity was monitored in the media for 72 h. As shown in figure 4A both cell lines secreted similar amounts of active NA independent of the construct. The viability of Hi5 cells seemed to drop faster (Fig. 4B) after infection compared to Sf21 cells, however, this did not affect the total amount of secreted NA activity. Western blots of the expression kinetics of both constructs showed strong expression of the respective NA constructs (Fig. 4C, D) in both cell lines whereas the signal seemed to drop slightly for the Hi5 expression after 72 h. As Sf21 cells are lower maintenance than Hi5 cells all further NAs were expressed in Sf21 cells.

### Large scale expression of construct 2 and 4

**Figure 5:**
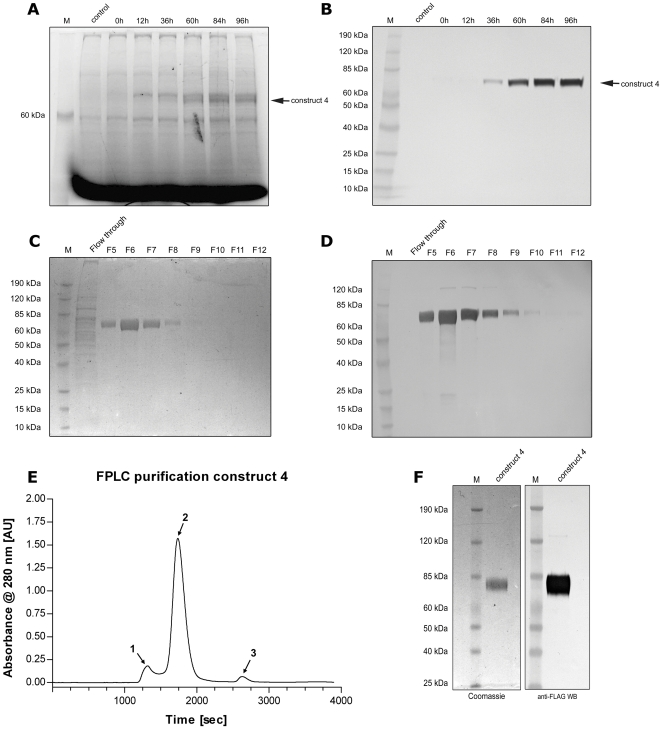
Large scale expression and purification exemplified by construct 4. To obtain sufficient amounts of NA, larger (5l) Sf21 cultures were infected and the respective NA was purified from the media. (A) UV protein imaging and (B) western blot of the 40,000 g media samples following the NA expression over 96 h. (C, D) Peptide eluted fractions from the anti-FLAG affinity matrix stained by Coomassie (C) or anti-FLAG western blot (D). (E) Gel filtration chromatogram of the FLAG-purified construct 4 recorded at 280 nm wavelength. (F) Purified construct 4 stained by Coomassie (left panel) or by anti-FLAG western blot (right panel).

Based on the results obtained from the optimization, large-scale Sf21 cultures were infected with the respective baculovirus stock to produce larger amounts of the different NA constructs. The NA expression and purification process was exemplified by the purification of construct 4. The accumulation of secreted NA over the time was monitored via its activity to ensure optimal yields (data not shown). In parallel, media samples were taken to track expression of construct 4 by PAGE and western blot (Fig. 5A, B). Although low in sensitivity, UV imaging of the gel (Fig. 5A) subsequently used for anti-FLAG western blot (Fig. 5B) showed that construct 4 was detectable. Anti-FLAG affinity chromatography, as an initial purification step, was already sufficient to purify the enzyme to virtual homogeneity as supported by the Coomassie stained gel (Fig. 5C) and the respective western blot (Fig. 5D). In addition, whereas the Coomassie stained gel showed the presence of a variety of proteins in the insect cell media (Fig. 5C; lane Flowthrough) the western blot indicated that all FLAG-reactive protein was pulled out of the media in a single run (Fig. 5D; lane FT) reflecting a total amount of 7 mg construct 4 (1.8 mg per litre culture). In order to further purify construct 4, the enzyme was concentrated and subjected to gel filtration chromatography. The chromatogram showed some high molecular weight impurities (Fig. 5E, peak 1) and a further peak (Fig. 5E, peak 3) that reflected the FLAG peptide used to elute construct 4 from the affinity column. The major peak (Fig. 5E, peak 2) contained the entire enzymatic activity of the sample (data not shown), and was concentrated down for subsequent characterization. The purity of construct 4 NA was estimated to be ∼99% based on the Coomassie stained gel and the corresponding anti-FLAG western blot (Fig. 5F). Construct 2 was also purified to homogeneity following the same protocol (data not shown), however, the total yield of construct 2 was usually only around 20–30% (0.5mg per litre) when compared to construct 4 indicating that the use of the Tetrabrachion stalk resulted in higher yields. The molecular weights of construct 2 and 4 were estimated by MALDI-ToF to be 54.6 kDa and 58.5 kDa, respectively. The N-terminal sequence of both proteins was validated by N-terminal sequencing (Table 1). Construct 3, although active in the media, was purified as two peaks in the gel filtration chromatography of which only the large molecular weight peak showed NA activity (data not shown). This result indicated that construct 3 was unstable during purification resulting in a mixture of active tetrameric NA and a non-active fraction of lower molecular weight potentially monomeric or dimeric NA.

**Table 1:** Biochemical properties of different NAs.

		Hokkaido virus	Construct 2	Construct 4
N-terminal sequencing		-	AEFDYKDDD…	AEFDYKDDD…
specific activity (pmol/sec/µg)		-	387±26.7	377±12.7
turnover number (molecules/sec/head)		-	79.4±5.5	77.2±2.6
*K*_m_ value for MUNANA (µM)		24.1±1.1	54.1±4.4	34.7±1.9
IC_50_	oseltamivir (nM)	3.72±0.45	12.5±1.9	8.67±1.33
	zanamivir (nM)	1.37±0.20	11.6±1.8	4.93±0.73
	peramivir (nM)	0.37±0.03	1.79±0.21	0.82±0.1
*K*_i_	oseltamivir (nM)	3.78±0.20	5.37±0.96	3.70±0.05
	zanamivir (nM)	1.28±0.07	3.85±0.75	3.19±0.22
	peramivir (nM)	0.50±0.03	1.42±0.04	0.51±0.13

The table shows the determined biochemical properties of the original Hokkaido virus NA and the artificial NAs derived from this virus. In addition, the IC_50_ and *K*_i_ values for the common NA-inhibitors oseltamivir, zanamivir, and peramivir are shown for all 3 NAs.

### Biochemical characterization of the purified artificial NAs

**Figure 6:**
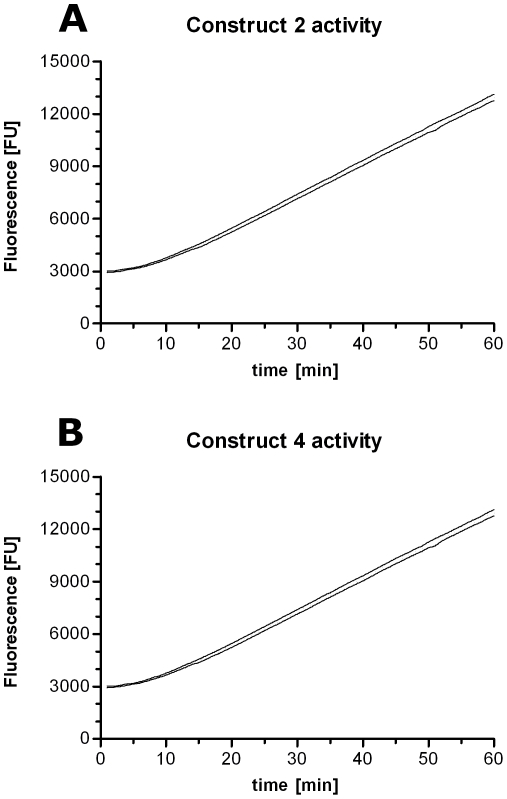
Maximal catalytic activity. To determine the specific activity of construct 2 (A) and 4 (B) a MUNANA activity assay was performed in excess substrate and at enzyme dilutions that allowed the enzyme reaction to remain linear over time. Data represents mean ± SEM (upper and lower line; mean is omitted for clarity) of 4 independent experiments performed in duplicate.

**Figure 7:**
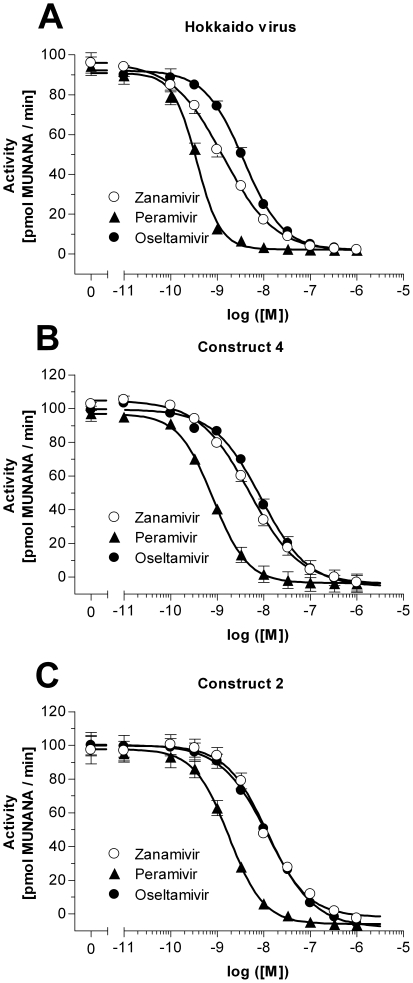
Determination of IC_50_s. To validate whether constructs 2 and 4 showed similar IC_50_ values for NA inhibitors, the enzymes (B, C) as well as the original Hokkaido virus (A) were incubated with increasing concentrations of oseltamivir, zanamivir, and peramivir. Data represents mean ± SEM of 4 independent experiments performed in duplicate.

**Figure 8:**
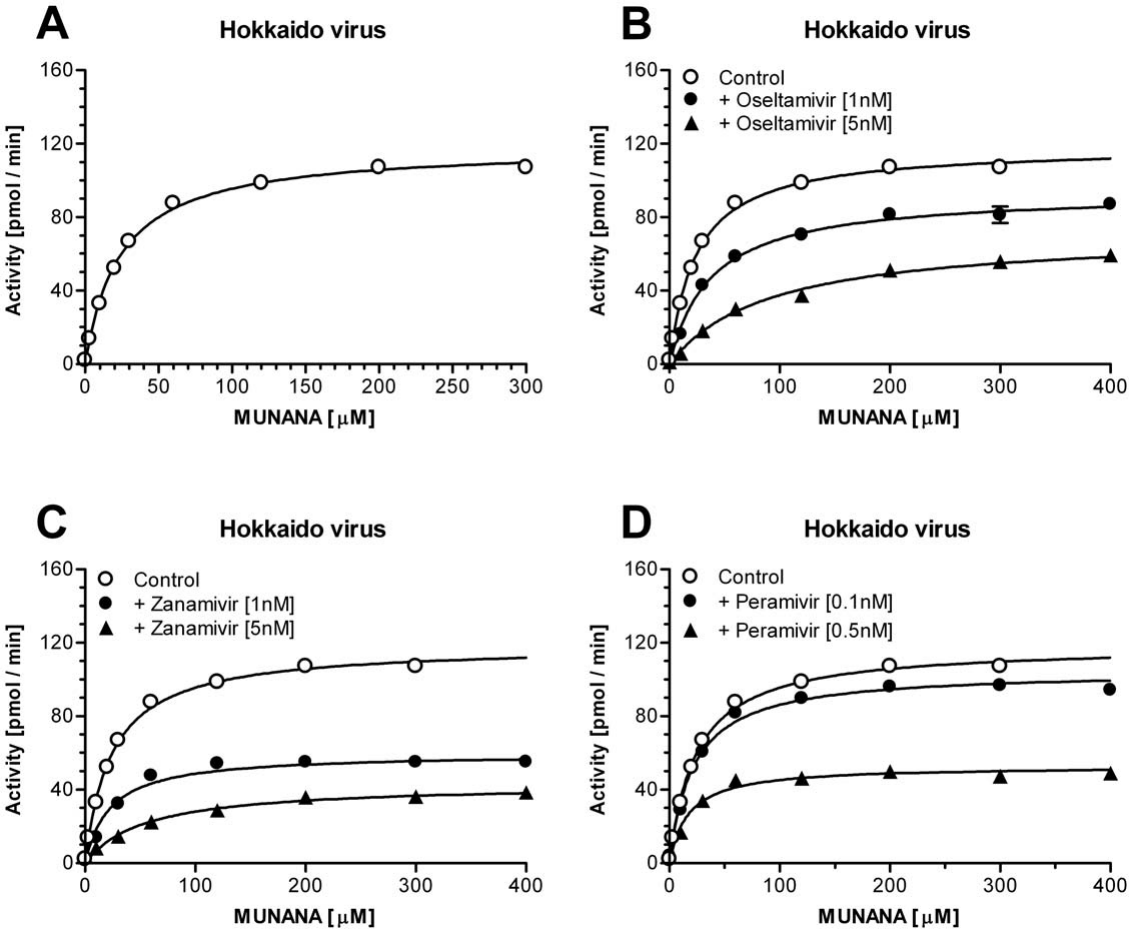
Determination of *K*_m_ and *K*_i_ values for the Hokkaido virus. To determine the *K*_m_ value for MUNANA as well as the *K*_i_ values for the three NA-inhibitors for the original Hokkaido virus, virus particles were incubated with increasing concentrations of substrate in the absence (A) or presence of oseltamivir (B), zanamivir (C), or peramivir (D). Data represents mean ± SEM of 4 independent experiments performed in duplicate.

**Figure 9:**
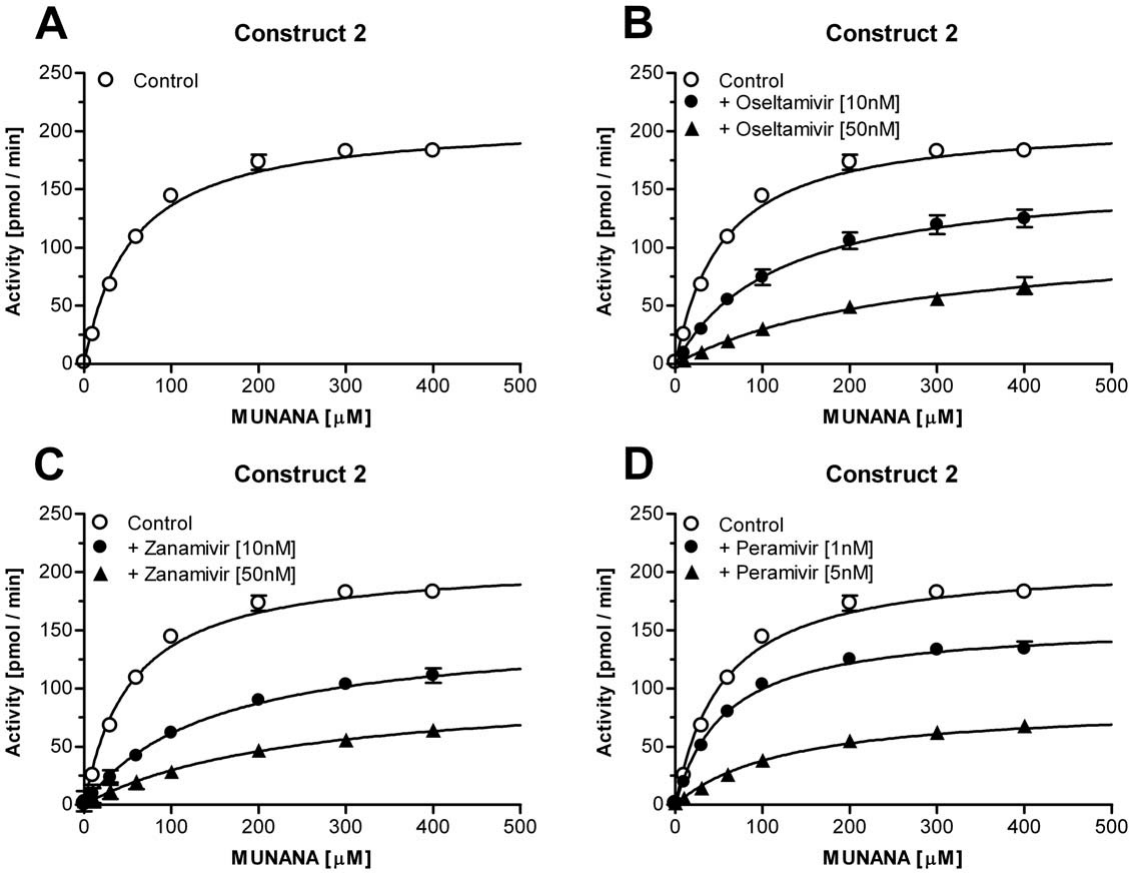
Determination of *K*_m_ and *K*_i_ values for construct 2. To determine the *K*_m_ value for MUNANA as well as the *K*_i_ values for the three NA-inhibitors for construct 2, purified enzyme was incubated with increasing concentrations of substrate in the absence (A) or presence of oseltamivir (B), zanamivir (C), or peramivir (D). Data represents mean ± SEM of 4 independent experiments performed in duplicate.

**Figure 10:**
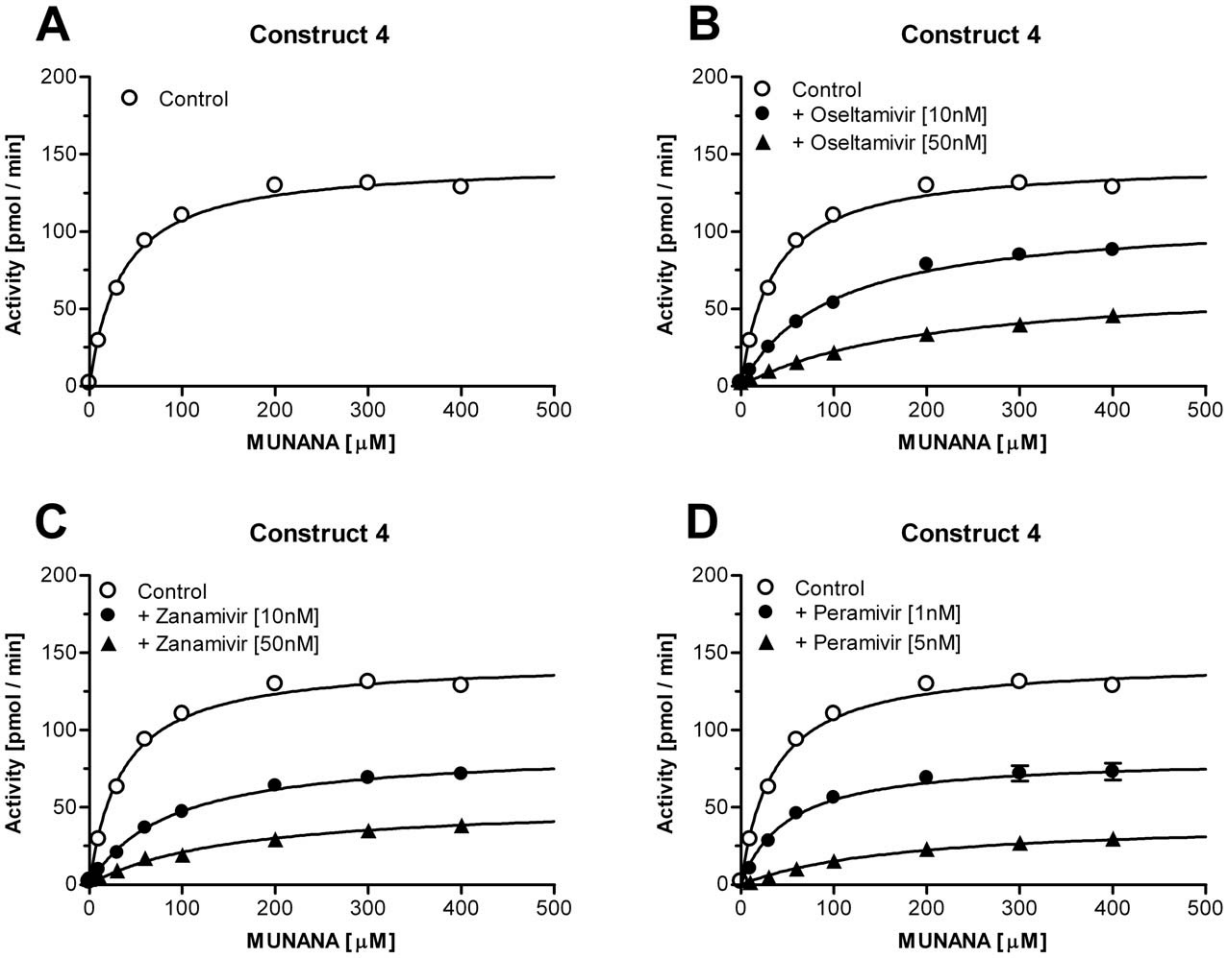
Determination of *K*_m_ and *K*_i_ values for construct 4. To determine the *K*_m_ value for MUNANA as well as the *K*_i_ values for the three NA-inhibitors for construct 4, purified enzyme was incubated with increasing concentrations of substrate in the absence (A) or presence of oseltamivir (B), zanamivir (C), or peramivir (D). Data represents mean ± SEM of 4 independent experiments performed in duplicate.

To determine the specific activity of the purified constructs 2 and 4 the enzymes were subjected to a 4-Methylumbelliferyl N-acetyl-a-D-Neuraminic acid (MUNANA) activity assay in the presence of excess substrate immediately after purification. Figure 6 shows the accumulation of fluorescent product over time for constructs 2 and 4. Based on the linear slope of both curves (FUs/sec), a 4-Methylumbelliferone (MU) standard, and the concentration of the enzyme in the assay, a specific activity of 387±26.7 and 377±12.7 pmol/sec/µg was calculated for construct 2 and construct 4 respectively (Table 1). To test whether the purified enzymes show comparable characteristics to the original viral NA, the *K*_m_ value for MUNANA as well as the IC_50_ values for the NA-inhibitors were determined. To calculate the IC_50_ values the Hokkaido virus and both recombinant enzymes were pre-incubated with increasing concentrations of NA inhibitors, oseltamivir, zanamivir and (1S,2S,3S,4R)-3-[(1S)-1-acetamido-2-ethyl-butyl]-4- (diaminomethylideneamino)-2-hydroxy-cyclopentane- 1-carboxylic acid (peramivir). The maximal observed catalytic rate for each reaction (FU/min) was measured and plotted against the respective inhibitor concentration. The IC_50_ values for oseltamivir, zanamivir, and peramivir were 3.72 nM, 1.37 nM, and 0.37 nM for the original Hokkaido virus (Fig. 7A). For construct 2 the IC_50_s were 12.5 nM, 11.6 nM, and 1.79 nM (Fig. 7B), whereas the values calculated for construct 4 were slightly lower, 8.67 nM, 4.93 nM, and 0.82 nM (Fig. 7C; Table 1). To investigate whether the exchange of the stalk had influenced the affinity towards the NA substrate, *K*_m_ values for the Hokkaido virus and both recombinant enzymes were determined by incubating the NAs with increasing concentrations of MUNANA in the absence or presence of NA-inhibitors oseltamivir, zanamivir, and peramivir (Fig. 8, 9, 10). The *K*_m_ value for the Hokkaido virus NA was 24.1 µM whereas the values for the recombinant enzymes were 54.4 µM and 34.7 µM for construct 2 and construct 4, respectively (Table 1). The *K*_i_ values for oseltamivir, zanamivir, and peramivir derived from the obtained *K*_m_ of the three NAs and the concentration of the respective NA inhibitor are summarized in Table 1. Construct 4 showed similar *K*_i_ values for oseltamivir, zanamivir, and peramivir (3.70 nM, 3.10 nM, 0.51 nM) compared to the Hokkaido virus (3.78 nM, 1.28 nM, 0.5 nM) whereas construct 2 had slightly elevated values (Table 1).

## Discussion

Despite seasonal influenza being responsible for a world-wide death toll of more than 250,000 per year, the availability of anti-viral therapies and vaccines have reduced the public concern about this common viral disease. However, this dramatically changed with the rapid spread of ‘swine flu’ and the world-wide emergence of the Tamiflu-resistant seasonal A(H1N1) H_274_Y human influenza. In the light of these incidents it is evident that understanding the emergence of resistance, the need to quickly develop sufficient doses of vaccine, and the development of new antiviral drugs are vital for the effective control of influenza. These goals would be greatly facilitated by the availability of larger amounts of stable and soluble NA making a robust, reliable, and universal expression system for this enzyme highly desirable. This publication describes a generic system that expresses highly soluble NA with biochemical and immunological properties close to the original virus NA. The production of purified NA may allow its use for various applications including high throughput screening for small molecule inhibitors, generation of anti-NA antibodies, binding assays, serotyping, protein crystallisation, or even vaccination.

The influenza stalk domain, although its sequence is poorly conserved, can be a major contributor to the stability of the homotetrameric structure of NA. Others have shown that secreted NA with a slightly truncated transmembrane domain resulted in the formation of monomeric, dimeric and tetrameric NA [12], [15]. These results suggest that the replacement of the NA stalk by more stable and rigid domains that are known to form parallel homotetrameric structures could promote greater NA stability. This approach was first formulated by Fiers et al. for influenza NA[20] and its feasibility to promote homotetramerization has been shown for proteins such as the influenza M2 [31] or human p53[32]. These publications made use of the yeast transcription factor GCN4 as a heterologous domain to promote tetramerization. Natural GCN4 forms parallel homodimers, however slight modifications of the residues in the dimer interface result in the stable formation of homotrimers or tetramers making this protein a very versatile tool[21]. As GCN4 has been shown to successfully promote tetramerization it was used as an artificial stalk for construct 1 and 2 described in this work. In general, domains that form parallel homotetramers are rare and a literature search revealed only artificial domains based on GCN4[33], [34] as well as two further natural domains, namely the tetramerization domain from the vasodilator-stimulated phosphoprotein (VASP)[35], [36], [37] and the protein Tetrabrachion from the deep sea archae bacteria *Staphylothermus marinus* [22], [23]. Based on its structural properties to form a straight parallel homotetramer[23], [36] and its stability even at high temperature[22], [23] the tetramerization domain from Tetrabrachion was used as the basis for constructs 3 and 4. To facilitate purification of the recombinant NA a FLAG tag was introduced N- (construct 2 and 4) or C-terminal from the tetramerization domain (construct 1 and 3). FLAG was chosen as it consists of only 8 residues and can increase the solubility of fused proteins [19], [38]. To enable purification from the media, the NA sequence was cloned downstream from a melittin signal peptide (MSP) sequence allowing recombinant proteins to be secreted from insect cells[18], [28].

Previously it was shown that even small modifications of the NA stalk are able to modulate the activity of the enzyme[27]. Contrary observations have been reported with respect to the introduction of a FLAG tag into the NA stalk domain. Some reports showed that the introduction of the affinity tag does not affect NA activity[39], [40] whereas Castrucci and co-workers reported a negative impact of this stalk modification on the catalytic rate[24]. In agreement with the latter report we found that the insertion of the FLAG tag in close proximity to the NA head had a strong destabilizing effect on the enzyme's tetrameric structure. This negative impact was reflected by the very low NA activity of construct 1 and the inability to purify construct 3 in a homogenous tetrameric state. This dissociation into catalytically inactive monomeric and dimeric NA due to the instability of the NA tetramer is a phenomenon that has been already described for recombinant NA[12], [15]. In striking contrast, construct 4 was stable, suggesting that the presence of FLAG in close proximity to the NA head disturbs the assembly of functional tetramers. A potential reason for the destabilizing effect might be the accumulation of 20 (4 FLAG tags) negatively charged aspartic acids in the tetrameric state resulting in electrostatic repulsion. The fact that construct 3 can be expressed as a functional protein in contrast to construct 1 suggests that the rigid Tetrabrachion stalk domain is able to confer a higher level of stability compared to the GCN4-pLI domain used for constructs 1 and 2. This view is supported by the fact that under natural conditions Tetrabrachion is stable at 130°C[22], [23]. This data suggests that in agreement with work published by Xu and co-workers[37] the affinity tag should be placed at the N-terminus of the recombinant NA to avoid interference with the formation of functional homotetramers. Moreover, the presence of 4 single N-terminal FLAG tags in the NA-tetramer seemed to result in a very high avidity as the entire FLAG reactive NA was pulled out of the media in a single run making this purification approach highly efficient and powerful.

The data from the optimization of the expression system shows that the secreted NAs (constructs 2 and 4) are highly active and soluble as the activity in the media was not decreased by ultracentrifugation, in contrast to membrane bound NA expressed in insects cells[16]. In addition, both enzymes retained their activity in insect cell media for weeks, even at room temperature (data not shown) underlining their improved stability. Nevertheless, the recombinant NAs were still vulnerable to low pH with construct 3 being less stable than construct 2 and 4. As the Tetrabrachion stalk used for construct 3 and 4 has been shown to be stable at a pH range from 1–11.5[22] the susceptibility of all artificial NA constructs to low pH is presumably due to the pH sensitivity of the NA head in agreement with published work[29], [30].

The optimization of expression conditions indicated that neither increasing the MOI beyond 1 nor expressing the recombinant NAs in Hi5 cells which require more maintenance than Sf21 insect cells increased the yields of soluble NA, in contrast to published results by others[37]. These results show that the NA expression system described here can produce high yields of NA. Milligram amounts of construct 4 NA per litre of insect cell culture were obtained from large scale expressions without obvious destabilization of the NA tetramer (based on size exclusion chromatography) or breakdown products (based on SDS-PAGE and western blot) as has previously been the case[12], [15], [16]. Compared to construct 4, construct 2 resulted in lower yields favouring the Tetrabrachion-based system for the expression or larger quantities of NA. The predicted molecular weights of secreted constructs 2 and 4 are 49.1 kDa and 51.2 kDa, respectively, however, when analysed by MALDI-ToF a slightly higher molecular weight of 54.6 kDa and 58.5 kDa was observed. The additional molecular weight of 5.5 kDa for construct 2 and 7.3 kDa for construct 4 as well as the broad shape of the band in the SDS-PAGE suggest that both enzymes are glycosylated, a common phenomena for NA[12], [13], [15]. Glycosylation of recombinant proteins expressed in insect cells are of a simpler high-mannose type compared to proteins expressed in mammalian cells[41] with an average molecular weight of approximately 1.3 kDa[41], [42]. The observed increase in molecular weight of 5.5 kDa and 7.3 kDa suggests 4 and at least 5 glycosylation sites for construct 2 and construct 4 respectively. Although the exact numbers of glycosylation sites for both enzymes have not been determined yet, these results are in good agreement with the predicted number of glycosylation sites being 4 for construct 2 and 5–6 for construct 4.

As a first step to biochemically characterize construct 2 and 4, the specific activity of both recombinant NAs was measured in a standard MUNANA activity assay. To determine the maximal catalytic rate with highest accuracy these assay were performed in the presence of excess substrate (1 mM) and at an enzyme dilution that allowed linear reaction conditions for an adequate time interval. Whereas former publications[15], [16], [43] needed to add high pH buffer to the enzymatic reaction in order to increase the MU fluorescence (thereby stopping the reaction), modern fluorescence plate readers allow the continuous recording of the catalytic conversion of MUNANA[5]. The specific activity based on the first derivative of the recorded enzymatic reaction (FU/sec) and a MU standard was determined to be 387 and 377 pmol/sec/µg that converts to a turnover number of 79 and 77 substrate molecules per second per NA tetramer. These values are slightly lower than values reported by others for example 870 pmol/sec/µg for N9 G70C NA[43]. However, it should be taken into account that these calculations rely on many variables e.g. the estimation of the protein concentration or the preparation of purified NA for example via proteolytic cleavage that might already affect the determined activities. Nevertheless, these values suggest that the nature of the artificial stalk did not seem to influence the specific activity under maximum substrate conditions. This result was not anticipated as the yeast GCN4 domain forms a slightly left-handed coiled coil structure whereas the Tetrabrachion domain forms a nearly parallel bundle[36]. Although the maximal catalytic activity was not affected, there were differences between the original virus NA and constructs 2 and 4 with respect to the *K*_m_ values for MUNANA. The native Hokkaido virus NA had a *K*_m_ value of 24.1 µM in good agreement with other influenza A virus strains[4]. In contrast the estimated *K*_m_ value for construct 2 was more than twice as high (54.1 µM) whereas construct 4 showed a *K*_m_ value that was closer to the original NA (34.7 µM). As all three enzymes share the same NA head these results might indicate that the slightly different twisting of the stalks[36] affected the positioning of the 4 monomers towards each other and as a consequence the observed affinity towards the NA substrate. It would be interesting to compare these results with an NA construct based on the VASP tetramerization domain that forms a right handed coiled-coil structure[36]. Unfortunately there has been no data published describing the biochemical properties of these recombinant enzymes[37], [44].

The decreased affinity for the NA substrate MUNANA was also reflected in the observed IC_50_ values for the NA inhibitors oseltamivir, zanamivir, and peramivir. Whereas the Hokkaido virus NA showed IC_50_ values in the range observed for various influenza A viruses[45] both recombinant enzymes, especially construct 2, showed increased IC_50_ values for all 3 drugs. These findings were supported by the *K*_i_ values derived from the enzyme activity in the presence of constant NA inhibitor. The inhibitory constant takes into account the decreased affinity for the substrate MUNANA in the absence of drugs making the *K*_i_ values a more reliable comparison between the original Hokkaido virus NA and the two recombinant enzymes. The *K*_i_ values for all three inhibitors for construct 4 were close to the ones obtained for the original virus NA, whereas construct 2 showed generally higher values. These results, in combination with the observed higher *K*_m_ value for construct 2, indicate that the replacement of the natural NA stalk by the yeast GCN4 stalk did affect the biochemical properties of the NA homotetramer, presumable by the twisted left-handed coiled-coil structure that has been reported for this domain[36].

In summary, this publication describes the construction and characterization of a generic baculovirus expression system for influenza NA that allow the production and straight forward purification of milligram amounts of NA from cell culture media. The results presented here indicate that the use of the Tetrabrachion tetramerization domain from the deep sea archae bacterium *Staphylothermus marinus* confers higher stability to the expressed and secreted NA than the yeast GCN4-pLI stalk. In addition, the Tetrabrachion-based recombinant NA is closer to the original viral NA with respect to its biochemical characteristics than the GCN4-based NA, making this system the preferred choice wherever stable and active NA is needed. The purified NA is a good candidate for biochemical applications ranging from high throughput screening for small molecule NA-inhibitors, production of monoclonal anti-NA antibodies, diagnostic applications, crystallisation studies or the generation of protein vaccines.

## Materials and Methods

### Materials

MUNANA was obtained from Carbosynth (Compton, Berkshire, UK). Neuraminidase inhibitors zanamivir, oseltamivir, and peramivir were synthesized at GlaxoSmithKline (Stevenage, UK). All other chemicals including MU were of analytical grade and purchased from Sigma-Aldrich (Castle Hill, Australia). FLAG peptide was ordered from Peptide2.0 (Chantilly, VA, USA). Monoclonal anti-FLAG M2 antibody was produced and purified at CSIRO, (Parkville, Australia). NAs were purified in Tris buffer [25 mM] pH 6.5 containing 10 mM Ca^2+^, and 0.02% azide (TBA). For activity assays NAs were diluted in TBA/BSA with a final concentration of BSA of 0.25 mg/ml (IC_50_ determinations) or 1 mg/ml (specific activity), respectively.

### Cells, virus, and media

Sf21 (*Spodoptera frugiperda*) and Hi5 (*Trichopulsia ni*) insect cells, cultured in SF 900 II (Invitrogen, Carlsbad, CA) or Express Five (Invitrogen) serum free medium, were used for baculovirus amplification and protein production. Influenza virus strain A/Hokkaido/15/02 H1N1 (hereafter referred to as Hokkaido, [45]) was amplified in Madin-Darby Canine Kidney (MDCK) cells propagated in maintenance medium (1∶1 mix of MEM media pH 6.8/L15 media pH 7.6, sodium bicarbonate [0.028%], HEPES pH 6.8 [20 mM], Fungizone [1 µg/ml], PenStrep/Glutamine).

### Construction of recombinant NA

Sequence construction and alignment was performed using Vector NTI (Invitrogen, Carlsbad, CA). cDNA was obtained by RT-PCR from influenza virus strain Hokkaido. The sequence encoding the NA head and a residual fragment of the original NA stalk was amplified using the primers Hokkaido-Stalk/Hokkaido-Head as shown in Table 2. The sequences for the artificial stalks including FLAG-tag were synthesized by GeneART (Regensburg, Germany). The artificial stalks were fused to the NA head via PCR using the relevant construct primer shown in Table 2. The PCR products were cloned into the transfer vector pFastBac downstream of the coding sequence for the MSP responsible for the secretion of the expressed protein[18]. As a control for some experiments, a baculovirus encoding the full length Hokkaido NA including the natural stalk as well as the transmembrane domain was generated. In the absence of a secretion signal the respective NA was expressed as membrane bound enzyme. Each DNA sequence was verified by sequencing (Micromon, Victoria, Australia). Bacmids and baculovirus particles were constructed using the Bac-to-Bac system (Invitrogen, Carlsbad, CA) according to the manufacturer's protocols.

**Table 2:** Primers used to amplify and fuse artificial stalk and NA head.

Name	direction	Sequence
Construct 1	forward	5-GCATCGGAATTCCTGAAGCAGATCGAGG-3
Construct 2/4	forward	5- GCATCGGAATTCGACTACAAGGACGACG-3
Construct 3	forward	5- GCATCGGAATTCGGTTCCATCATCAACG-3
Hokkaido-Stalk	forward	5-CAACACCAATGTTATTGCTGGAAAGGAC-3
Hokkaido-Stalk	reverse	5-CACCAATGTTATTGCTGGAAAGGACAAAACTTC-3
Hokkaido-Head	reverse	5-GCTCTAGATTACTACTTGTCAATGGTGAATGGCAAC-3

All primers are shown in 5′ to 3′ direction. The underlined nucleotides represent the restriction sites (EcoRI, XbaI) used for subsequent cloning into pFastBac-MSP.

### Expression and purification of recombinant NAs

Recombinant NA-constructs were expressed in Sf21 insect cells (Invitrogen, Carlsbad, CA) cultured in SF 900II serum free medium (Invitrogen, Carlsbad, CA). Cells were infected at a density of 2×10^6^/ml with an MOI of 1. Secreted NA activity in the media and cell viability were assessed every day. 4 days post-infection cells, cell debris, and baculovirus were spun down (40,000 g, 1 h, 4°C) and the cleared supernatant was filtered through 1.2 µm and 0.22 µm filters. Azide and the protease inhibitor E-64[46] were added to a final concentration of 0.02% and 1 µM, respectively. The cleared supernatant was run overnight through a 50 ml anti-FLAG affinity column (Mini-Leak Low; Kem-En-Tec A/S, Copenhagen, Denmark) at 4°C and bound protein was eluted by TBA buffer containing 0.25 mg/ml FLAG peptide (DYKDDDDK). The NA containing fractions, identified by their activity, were pooled, and concentrated down to 1 ml by using 10 kDa cutoff spin concentrator (Millipore Amicon Ultra-4). Subsequently, the concentrated NA sample was further purified via gel filtration on a Superose12 column (GE Healthcare, Rydalmere, NSW, Australia) using a BioLogic DuoFlow System equipped with a QuadTec UV-Vis detector (Bio-Rad, Gladesville, NSW, Australia). The NA-containing fractions were pooled and concentrated down for further analysis.

### SDS-PAGE and Western blotting

SDS-PAGE was performed using NuPAGE (Invitrogen, Carlsbad, CA) or iGels (NuSep, Frenchs Forest, NSW, Australia) precast gels according to the manufacturer's protocols. Protein bands were visualized by Coomassie Brilliant Blue (NuSep), Silver stain[47], or UV by using the protein stain NUView, which is already incorporated in iGels. Western blots were performed using the iBlot system (Invitrogen, Carlsbad, CA) according to the manufacturer's protocol. Transferred FLAG-tagged proteins were visualized as described elsewhere[48], briefly, the nitrocellulose membrane was blocked using 1% Casein/PBS and subsequently incubated with anti-FLAG mAb in 1% Casein/PBS (1/1000). After washing, membranes were incubated with secondary antibody (anti-mouse-HRP; ImmunoBioScience, Mukilteo, WA) in 1% Casein/PBS (1/10000). Immunoreactive proteins were detected using TMB (3,3′,5,5′-Tetramethylbenzidine) as described[48] except that dextran sulphate [1%] was added directly to the TMB substrate solution as it was found to improve colour development.

### N-terminal sequencing

The N-terminal sequences of the purified proteins were determined by automated Edman degradation in a Hewlett-Packard G1000A sequencer as described elsewhere[49].

### Neuraminidase activity assay

The activity of expressed NAs was measured based on published protocols using the fluorescent NA substrate MUNANA[50], [51] with the modification that fluorescence was monitored continuously. Briefly, purified enzyme, cleared cell culture supernatant, or influenza virus particles were diluted in TBA. The enzyme reaction was initiated by adding 2-fold concentrated acetate buffer [200 mM, pH 5.5] containing Ca^2+^ [10 mM] and MUNANA [200 µM]. Enzyme activity was measured by continuously monitoring the increasing fluorescence of the reaction product MU (excitation: 355 nm, emission 460 nm) in black 96 well plates (PerkinElmer, California, USA) for 60 min at 37°C. Fluorescence was recorded using a Fluostar OPTIMA plate reader (BMG Labtech, Mornington, Australia) and data was subsequently analysed using MARS data analysis software (BMG).

#### pH sensitivity

To determine whether low pH has an impact on the activity of the recombinant NAs 50 µl of constructs 2–4 in SF 900II media were diluted ^1^/_20_ in glycine buffer pH 2.8 [200 mM] followed by subsequent neutralisation with Tris buffer pH 8.7 [1 M] after 1 min, 15 min, and 30 min. The remaining NA activity was determined by standard MUNANA assay as described above.

#### IC_50_ determination

In order to determine the IC_50_ values for zanamivir, oseltamivir and peramivir for the original virus (Hokkaido) as well as for the recombinant soluble NA constructs, the concentration of NA was titrated to ensure linearity of the enzyme reaction over time. The respective NA dilution in TBA/BSA was pre-incubated with increasing concentrations of NA inhibitors for 30 min at 37°C. The enzyme reaction was started by the addition of MUNANA-containing substrate buffer as described above. The maximum rate of enzyme activity (FU/min) was calculated for each reaction and graphed against the respective inhibitor concentration. IC_50_ values were defined as the inhibitor concentration that caused a 50% reduction of the maximum catalytic rate of the no-drug-control.

#### Specific activity

Specific activities of purified NAs were determined in the presence of excess MUNANA [1 mM final concentration] to avoid substrate depletion. The maximum activity of each enzyme (FU/min) was calculated from three different enzyme concentrations. A MU standard was included to allow the conversion of FU into molarities.

#### Kinetic parameters

In order to determine the *K*_m_ values for the original virus NA and the recombinant soluble NAs, the enzymes were incubated with increasing concentrations of MUNANA (3 µM–600 µM) in the absence and presence of the NA inhibitors zanamivir, peramivir, or oseltamivir for 30 min at 37°C. The enzyme reaction was started by adding substrate-buffer and the reaction was monitored for 60 min at 37°C as described above. Data was analysed using MARS data analysis software (BMG). The highest observed enzyme rate (FU/min) was plotted against the substrate concentration. The *K*_m_ values for MUNANA as well as the *K*_i_ values for the NA inhibitors zanamivir, oseltamivir, and peramivir were determined by PRISM 5 (GraphPad Software, La Jolla, USA) using the non-linear regression for mixed-mode inhibition.

## Supporting Information

Figure S1 **Expression kinetic of construct 1-4.** Anti-FLAG western blots of Sf21 supernatants without infection (72 h control) or infected with construct 1-4 (MOI 1). Samples of infected cells were taken at the indicated time points.
(TIF)

Table S1 **Names of influenza strains included in the initial alignment.** The table lists the names and the NA/HA composition of the 43 influenza strains from 1918-2007 which NA sequences (N1-N9) were included in the initial multisequence alignment shown in figure 1.
(DOC)
