# Supplementary figures and images for: A Generic System for the Expression and Purification of Soluble and Stable Influenza Neuraminidase
Source: PLoS One. 2011 Feb 7;6(2):e16284. doi: 10.1371/journal.pone.0016284 (PMC3034727; doi:10.1371/journal.pone.0016284)

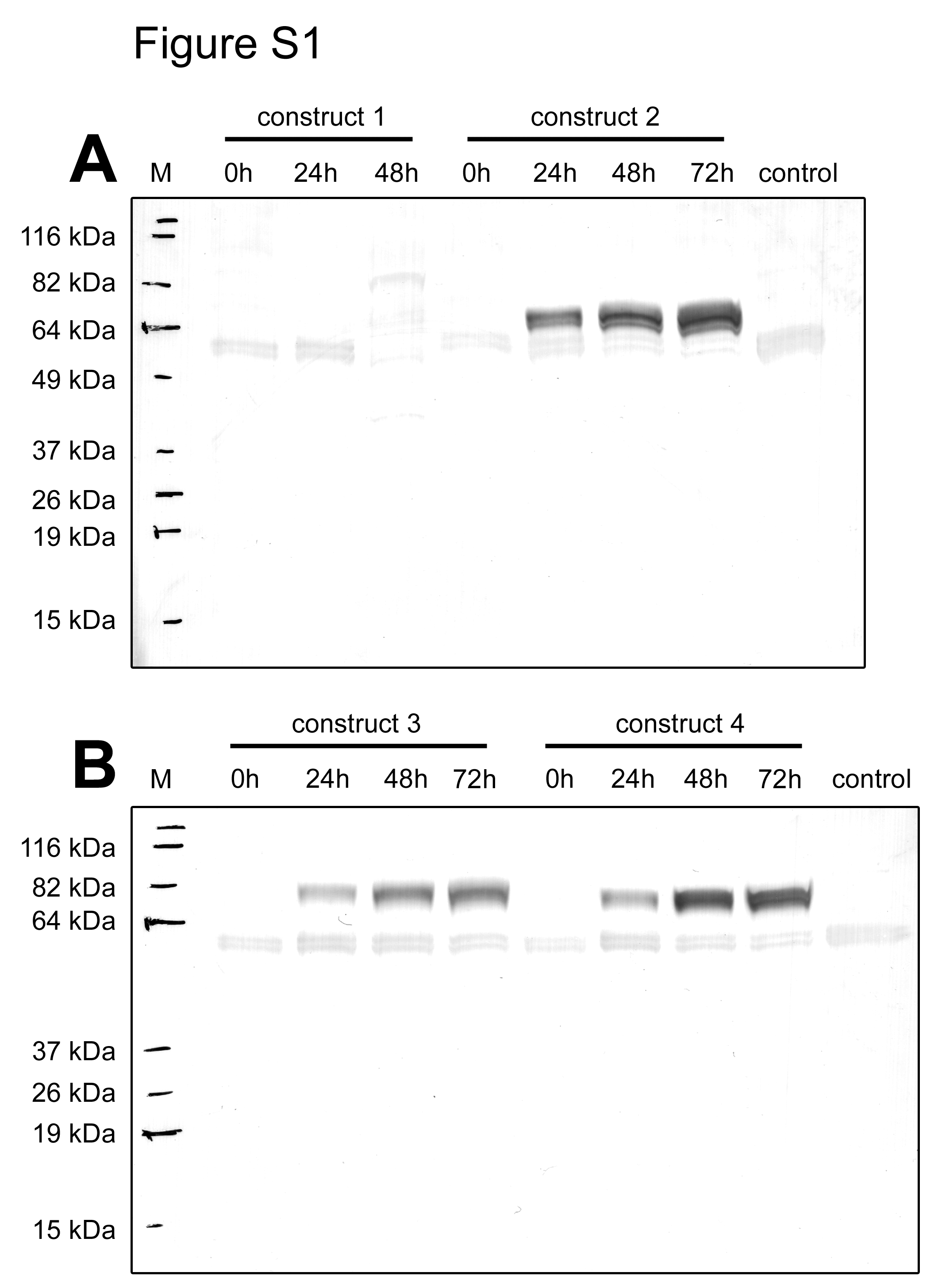

Supplement: Figure S1 — Expression kinetic of construct 1-4. Anti-FLAG western blots of Sf21 supernatants without infection (72 h control) or infected with construct 1-4 (MOI 1). Samples of infected cells were taken at the indicated time points. (TIF) [file pone.0016284.s001.tif]
