# Supplementary material for: A Generic System for the Expression and Purification of Soluble and Stable Influenza Neuraminidase
Source: PLoS One. 2011 Feb 7;6(2):e16284. doi: 10.1371/journal.pone.0016284 (PMC3034727; doi:10.1371/journal.pone.0016284)
Supplement: Table S1 — Names of influenza strains included in the initial alignment. The table lists the names and the NA/HA composition of the 43 influenza strains from 1918-2007 which NA sequences (N1-N9) were included in the initial multisequence alignment shown in figure 1. (DOC) [file pone.0016284.s002.doc]

Table S1

| **Neuraminidase (NA)** | **Hemagglutinin (HA)** | **influenza strain** |
| --- | --- | --- |
| N1 | H1 | A/South Carolina/1918 |
| N1 | H1 | A/Wilson-Smith/1933 |
| N1 | H1 | A/Puerto Rico/8/34/Mount Sinai |
| N1 | H1 | A/Fort Monmouth/1/47-MA |
| N1 | H1 | A/Leningrad/1/1954 |
| N1 | H1 | A/New Jersey/8/1976 |
| N1 | H1 | A/USSR/90/1977 |
| N1 | H1 | A/Kiev/59/1979 |
| N1 | H1 | A/Chile/1/1983 |
| N2 | H2 | A/Japan/305/1957 |
| N2 | H2 | A/Leningrad/134/1957 |
| N2 | H2 | A/RI/5-/1957 |
| N2 | H2 | A/Singapore/1/1957 |
| N2 | H2 | A/Ann Arbor/6/1960 |
| N2 | H2 | A/Tokyo/3/1967 |
| N2 | H2 | A/Korea/426/1968 |
| N2 | H3 | A/England/878/1969 |
| N3 | H7 | A/turkey/Oregon/1971 |
| N3 | H3 | A/Bantam/Nanchang/9-366/2003 |
| N3 | H7 | A/chicken/British Columbia/GSC-human-B/04 |
| N4 | H8 | A/duck/Hokkaido/95/1981 |
| N4 | H9 | A/duck/Hokkaido/HY57/2005 |
| N4 | H8 | A/mallard/Alaska/708/2005 |
| N4 | H4 | A/gray teal/Australia/2/1979 |
| N5 | H12 | A/duck/Alberta/60/1976 |
| N5 | H8 | A/duck/Tsujuba/255/2005 |
| N5 | H6 | A/mallard/Netherlands/11/2007 |
| N5 | H6 | A/Shearwater/Australia/1/1972 |
| N6 | H16 | A/duck/Seberia/272/1998 |
| N6 | H1 | A/mallard/Alberta/42/1977 |
| N6 | H11 | A/duck/England/1/1956 |
| N7 | H10 | A/duck/Alaska/712/1991 |
| N7 | H7 | A/Netherlands/33/2003 |
| N7 | H5 | A/mallard/64650/2003 |
| N7 | H7 | A/chicken/FPV/Weybridge |
| N8 | H3 | A/equine/Miami/1/1963 |
| N8 | H3 | A/duck/Ukraine/1/1963 |
| N8 | H10 | A/quail/Italy/1117/1965 |
| N9 | H11 | A/duck/Memphis/546/1974 |
| N9 | H11 | A/duck/Chiba/7/2006 |
| N9 | H11 | A/mallard/Maryland/538/2002 |
| N9 | H11 | A/tern/Australia/G70C/1975 |
| N9 | H13 | A/whale/Maine/1/1984 |
